# Clinicopathological features and correlation analysis of male breast cancer

**DOI:** 10.1097/MD.0000000000034408

**Published:** 2023-07-28

**Authors:** Lei Xi, Jinxing Zhou, Yan Wu, Rong Rong

**Affiliations:** a Department of Pathology, The First Affiliated Hospital of Nanjing Medical University, Nanjing, China.

**Keywords:** East Asian, male breast cancer, retrospective study, SEER

## Abstract

To analyze and compare the clinicopathological characteristics of male breast cancer (MBC) among Chinese patients and those from East Asia and other regions. Clinicopathological data from 3 kinds of data sources, including 31 MBC patients in Jiangsu Provincial Hospital (JPH) from 2014 to 2021 in China, 20 literature data on East Asian MBC patients from 2014 to 2021, and 3102 MBC patients registered in the surveillance, epidemiology, and end results (SEER) database from 2014 to 2019, were collected and retrospectively analyzed. The average ages of first-diagnosis MBC patients in JPH and East Asian patients were 59.7 and 62.3 years old, respectively, which were younger than those of SEER patients (66.5 years old). Between East Asian and SEER patients, the status or rates of main breast cancer type, estrogen receptor, progesterone receptor, human epidermal growth factor receptor 2, breast subtype, and TNM stage were relatively close, and their differences were not statistically significant (*P* > .05). Differences were observed in chemotherapy, surgery, pathological grade, and lymph node positivity (*P* < .01). Furthermore, no statistically significant difference was observed between the JPH and East Asian patients (all *P* > .05). In JPH and SEER, linear regression relationships were observed between the lymph node positivity rate, tumor size, and histological grade. JPH and East Asian MBC patients were younger than SEER patients. Between East Asian and SEER patients, the status of the main breast cancer type, estrogen receptor, progesterone receptor, human epidermal growth factor receptor 2, breast subtype, and TNM stage were similar, but there were differences in chemotherapy, surgery, pathological grade, and lymph node positivity. The findings of this study should prove to be helpful to deepen our understanding of East Asian MBC.

## 1. Introduction

Male breast cancer (MBC) is a rare malignant tumor with an incidence of <1% in malignant tumors, but its incidence has been increasing in recent years.^[1]^ MBC has a poorer prognosis, and its 5-year survival rate and overall survival (OS) rates are approximately 10% and 15% lower than those of female patients, respectively. After considering the differences in clinicopathological factors, the mortality rate of MBC after diagnosis was still higher than that of the female patients.^[2]^ Given the small number of MBC cases, conducting effective prospective studies is difficult. Clinical diagnosis and treatment mainly refer to the treatment mode for breast cancer in women. This study aimed to compare the clinicopathological characteristics of MBC patients among Jiangsu Provincial Hospital (JPH), East Asian, and surveillance, epidemiology, and end results (SEER) patients and retrospectively analyze the relationship between different patients and the correlation and regression relationship between some index factors. This study selects SEER data (including different races) as a reference or standard for comparison with data from East Asia and JHC.

## 2. Materials and methods

### 2.1. General materials

First, clinicopathological data from 3 sources, including 31 MBC patients in JPH from 2014 to 2021 in China, 20 literature data on East Asian MBC patients in 2014 to 2021, and 3102 MBC patients registered in the SEER database from 2014 to 2019, were collected. A comparison was then performed on the clinicopathological data, which included age at first diagnosis, breast cancer type, estrogen receptor (ER), progesterone receptor (PR), human epidermal growth factor receptor 2 (HER2), surgery, chemotherapy (CH), breast subtype, histological grade, TNM stage, and lymph node positivity status.

### 2.2. Methods

This retrospective study adhered to institutional guidelines and was approved by the Institutional Review Board of the First Affiliated Hospital of Nanjing Medical University (IRB No. 2023-SR-126). The author selected the keywords “breast cancer” and “male” and searched the literature on East Asian MBC patients in the CNKI and Wanfang Data. A search method combining keywords and free words was used. In addition, using the keywords “MBC” and “MBC,” the authors searched for literature involving East Asian patients in PubMed, EMbase, and the Cochrane Library, with a publication period between 2014 and 2021. References to relevant papers were manually searched as a supplement. The inclusion criteria for the East Asian literature included in this study were as follows: male sex, breast as the primary site, publication time between 2014 and 2021, and East Asian origin of patients. The exclusion criteria were as follows: incomplete main data, such as those of ER, PR, and HER2; and patients from non-East Asian regions. The article selection process is shown in Supplementary Figure S1, http://links.lww.com/MD/J339.

### 2.3. Statistical analysis

SPSS software (version 23.0) was used for the statistical analysis. The data in this study are expressed as percentages or arithmetic means. Pearson *χ*^2^ test was used for comparisons between groups, and statistical significance was set at *P* < .05.

## 3. Results

Tables 1 to 3 summarize the data of MBC patients from JPH, 20 East Asian studies (18 in Mainland China, 1 each in Hong Kong, China, and South Korea),^[3–22]^ and the SEER database, respectively.

**Table 1:** Data of male breast cancer (MBC) in Jiangsu Provincial Hospital (JPH).

Age	Type	ER	PR	HER2	CH	Surgery	Subtype	HG	TNM	Node	T stage
62	ot	2+	2+	u	n	y	a	II	III	−	u
83	I	−	−	−	n	y	d	II	I	−	1
55	I	2+	1+	3+	y	y	b	III	IV	u	1
73	I	1+	2+	2+	y	y	b	III	III	+	2
74	ot	1+	2+	−	y	y	a	II	II	−	2
36	I	2+	2+	+	n	y	a	II	I	−	1
58	I	2+	2+	−	n	y	a	II	I	−	1
51	I	3+	+	−	n	y	a	II	I	−	1
80	I	3+	3+	2+	y	y	a	III	II	u	2
72	ot	3+	2+	2+	y	y	a	III	I	u	2
74	I	3+	+	2+	y	y	b	III	II	+	2
59	I	3+	3+	+	y	y	a	II	II	+	2
65	I	2+	+	3+	y	y	b	III	II	+	2
74	I	2+	2+	2+	y	y	a	III	III	+	2
49	I	3+	2+	−	y	y	a	II	II	+	2
67	I	2+	2+	+	n	y	a	II	I	−	1
55	ot	3+	3+	0	n	y	u	I	I	−	1
43	I	3+	2+	2+	y	y	a	III	III	u	u
50	ot	3+	3+	0	y	y	u	III	I	−	1
49	I	2+	2+	+	n	y	a	II	I	−	1
48	I	3+	3+	0	y	y	a	II	III	+	2
54	I	2+	2+	2+	n	y	a	I	I	−	1
38	I	2+	+	2+	n	y	a	II	I	−	1
75	I	2+	2+	0	y	y	a	II	II	−	2
57	I	2+	2+	−	y	y	a	III	II	+	2
53	I	3+	2+	−	n	y	a	II	I	−	1
67	I	3+	−	3+	y	y	b	III	III	+	2
49	I	3+	2+	−	n	y	a	II	I	−	1
66	I	3+	2+	+	y	y	a	II	II	+	2
53	I	2+	1+	−	n	y	a	II	I	−	1
61	ot	2+	2+	+	n	y	a	I	I	−	1
Total	25/6 (I/ot)	+30/−1	+29/−2	+3/−27/u1	y17/n14	y31/n0	23/5/0/1/2 (a/b/c/d/u)	3/17/11 (I/II/III)	15/9/6/1 (I/II/III/IV)	+10/−17/u4	15/14/2 (T_1_/T_2_/u)
%	80.6/19.5	96/4	93.6/6.4	10/90	54.8/45.2	100/0	79.3/17.2/0/3.4	9.6/54.8/35.5		37/67	51.7/48.3

a = HR+/HER2−, b = HR+/HER2+, c = HR−/HER2+, CH = chemotherapy, d = HR−/HER2−, HG = histologic grade, I = IDC (invasive ductal carcinoma), n = no, ot = other, u = unknown, y = yes.

Calculated from the data in Table 1, the median age of the 31 patients with JPH was 58 (36–83) years, and the average age was 59.7 years old. The main pathological type was invasive ductal carcinoma (IDC) in 25 cases (80.6%). The number of ER-positive, PR-positive, and HER2-positive results was 30, 29, and 3, respectively. All patients underwent surgery and 17 underwent CH. Regarding breast subtype, luminal A (HR+/HER2−) was observed in 23 cases. In terms of TNM staging, 15, 9, and 6 patients were in stage I, II, and III, respectively. Therefore, patients with stage 0 to II MBC accounted for approximately 77.4% (24/31) and those with stages III to IV accounted for 22.6% (7/31). The axillary lymph nodes were positive in 10 patients.

As presented in Table 2, the average age of MBC patients from 20 East Asian literature was 62.1 years. The main pathological type was IDC (83.4%), and the proportions of ER, PR, and HER2 were 92.2%, 87.7%, and 11.7%, respectively. The proportions of patients receiving CH and surgery were 53.9% and 92.9%, respectively. In terms of breast subtype, 606 cases (82.9%) were HR+/HER2− and 101 cases were HR+/HER2+. A total of 49 (12%) and 262 patients were diagnosed with pathological grade I and II MBC, respectively. In TNM staging, 485 patients (71.6%) were in stage 0 to II, and 192 patients were in stages III to IV. The proportion of positive axillary lymph nodes was 30.2%.

**Table 2:** Data on male breast cancer (MBC) in East Asian literature.

	Age	Type (I/ot/u)	ER (+/−/u)	PR (+/−/u)	HER2 (+/−/u)	CH (y/n/u)	Surgery (y/n/ u)	Subtype (a/b/c/d/u)	HG (I/II/III/u)	TNM (0–II /III–IV/u)	Node (+/−/u)
Wu^[3]^	63	23/14/3	34/2/4	34/2/4	4/26/10	15/25/0	39/1/0	25/4/0/1/10	u	26/8/6	12/28/0
Qin^[4]^	58	39/11/0	42/5/3	41/6/3	3/44/3	26/24/0	49/1/0	23/16/3/5/3	u	29/15/6	20/30/0
Li, H^[5]^	u	29/9/0	36/2/0	35/3/0	2/27/9	8/30/0	38/0/0	18/16/0/2	2/20/7/9	19/10/9	16/22/0
Zhang, Y^[6]^	63.7	22/3/0	23/2/0	23/2/0	5/20/0	13/12/0	23/2/0	16/5/0/0/4	4/10/9/2	17/8/0	9/16/0
He, C^[7]^	u	14/8/0	16/2/4	16/2/4	4/14/4	u	u	14/2/0/0/6	u	10/8/4	9/9/4
Zhang, X^[8]^	62.2	22/8/0	22/1/7	22/1/7	1/18/11	u	28/2/0	18/1/0/0/11	u	u	16/12/0
Mo^[9]^	u	132/16/0	139/9/4	136/12/4	11/137/3	92/60/0	152/0/0	128/8/0/0/14	u	144/38/0	67/85/0
Li, Y^[10]^	u	u	22/3/0	22/3/0	2/23/0	13/12/0	23/2/0	22/3/0/0/0	u	13/12/0	13/12/0
He, Y^[11]^	u	30/5/0	30/2/3	31/1/0	2/23/0	152/0/0	35/0/0	27/3/0/0	1/22/3/9	u	15/20/0
Shi^[12]^	u	25/3/0	24/4/0	24/4/0	3/25/0	23/5/0	22/0/6	23/1/3/0/1	1/6/21	u	23/5/0
Song^[13]^	u	35/4/0	36/3/0	36/3/0	0/37/2	u	37/2/0	35/0/0/2/2	8/26/5	u	3/36/0
Shao^[14]^	60.4	9/2/0	11/0/0	9/2/0	1/10/0	1/10/0	9/2/0	10/0/0/0/1	0/9/2	8/3/0	4/7/0
Wang, Y^[15]^	56	35/3/0	34/4/0	26/12/0	8/30/0	35/3/0	35/3/0	30/4/4/0/0	u	u	25/13/0
Ma^[16]^	62.3/0	33/10/0	40/3/0	36/7/0	3/40/0	16/27/0	35/8/0	36/3/0/1/3	u	32/11/0	10/33/0
Liu, Y^[17]^	u	7/3/0	7/0/3	7/0/3	2/6/2	5/5/0	10/0/0	6/1/0/0/3	u	4/5/1	5/5/0
Xu,w^[18]^	u	87/27/0	100/6/8	96/10/8	16/68/30	107/7/0	114/0/0	68/8/0/0/38	6/9/0	70/24/20	40/64/10
Ding, Y^[19]^	u	24/3/0	18/1/8	18/1/8	1/17/9	u	27/0/0	17/1/0/0/9	1/7/1	u	7/8/0
Sun^[20]^	u	34/10/0	40/4/0	37/7/0	2/42/0	27/17/0	40/0/0	25/16/0/3/0	1/30/13	23/21/0	16/28/0
Kwong^[21]^	64.5	116/16/0	100/6/26	84/15/33	14/39/78	22/82/28	107/25/0	36/5/0/3/88	20/41/20/51/0	77/18/20	30/64/38
Hong, J^[22]^	u	44/15/0	38/4/17	27/15/17	11/29/19	19/40/0	50/9/0	29/4/0/0/26	5/22/21/11	43/11/5	18/15/26
Total	62.1 (v)	788/155	931/79	873/123	93/699	447/382	873/67	606/101/7/17/210	49/262/97/72	485/192/67	358/828
%		83.4/16.6	92.2/7.8	87.7/12.3	11.7/88.3	53.9/46.1	92.9/7.1	82.9/13.8/1/2.3	12.0/64.2/23.8	71.6/28.4	30.2/69.8

a = HR+/HER2−, b = HR+/HER2+, c = HR−/HER2+, CH = chemotherapy, d = HR−/HER2−, HG = histologic grade, I = IDC, n = no, ot = other, PR = progesterone receptor, u = unknown, v = average, y = yes.

As shown in Table 3, among the 3102 MBC patients in the SEER study, the median age was 68 years (range, 15–85 years), and the average age was 66.7 years old. The main pathological type was IDC (85.9%). The positive proportions for ER, PR, and HER2 were 96.6%, 89.8%, and 13.7%, respectively. The proportions of patients receiving CH and surgery were 35.2% and 83.9%, respectively. In terms of breast subtypes, the proportion of HR+/HER2− was 40% and that of HR+/HER2+ was 12.9%. In TNM staging, patients in stages 0 to II accounted for 75.2%, and those in stages III to IV accounted for 24.8% (613/2476). Axillary lymph nodes were positive in 41% of patients.

**Table 3:** Pathological data of male breast cancer (MBC) in surveillance, epidemiology, and end results (SEER).

	Age (m/v)	Type (I/ot)	ER (+/−/u)	PR (+/−/u)	HER2 (+/−/u)	CH (y/n)	Surgery (y/n/ u)	Subtype (a/b/c/d/u)	HG (I/II/III/u)	TNM (0,I,II, III, IV/u)	Node (+/−/u)
Total	68/66.7	2666/436	2845/10/155	2632/299/171	384/2425/291	1092/2010	2602/489/11	2354/362/22/65/299	219/959/634/1285	6/846/1011/397/216/626	381/547/2129
%		85.9/14.1	96.6/3.4	89.8/10.2	13.7/86.3	35.2/64.8	83.9/0.161	84/12.9/0.8/2.3	12.1/52.8/34.9		41/59

a = HR+/HER2−, b = HR+/HER2+, c = HR−/HER2+, CH = chemotherapy, d = HR−/HER2−, HG = histologic grade, I = IDC, m = median, n = no, ot = other, PR = progesterone receptor, u = unknown, v = average, y = yes.

### 3.1. Comparison of the clinicopathological characteristics of 3 data sources

The above information showed that the average age of MBC patients from JPH was 59.7 years old, which is approximately 2 and 6 years younger than the average age of patients from East Asia (62.1 years old) (*P* < .01) and SEER studies (66.5 years old), respectively (*P* < .01).

For convenience of comparison, this study conducted the *χ*^2^ test (fourfold table) of the corresponding indicators of the 3 data sources, and the results are shown in Table 4. The results showed that some indicators were similar between East Asian and SEER patients (*P* > .05). For example, the main pathological type is IDC, with a rate of >80%. The positivity rates of ER, PR, and HER2 were approximately 92%, 86.7%, and 10%, respectively. The most common breast subtype was HR+/HER2−, accounting for >79% of the cases, followed by HR+/HER2+ (12.9%). The proportion of TNM stages 0 to II was >71%.

**Table 4:** Comparison of the features of male breast cancer (MBC) in surveillance, epidemiology, and end results (SEER), East Asian, and JPH databases.

	SEER (%)	^++^ *χ* ^2^	^++^ *P*	East Asian (%)	^*+^χ^2^	^*+^ *P*	JPH (%)
Type		3.75	.07		0.16	.22	
IDC	2666 (85.9)			778 (83.4)			25 (80.6)
Other	436 (14.1)			155 (16.6)			6 (19.4)
ER		0.96	.42		0.89	.35	
+	2845 (96.6)			931 (92.8)			30 (96.7)
−	100 (3.4)			79 (7.2)			1 (3.3)
PR		3.58	.07		0.98	.41	
+	2632 (89.8)			873 (87.6)			29 (93.5)
−	299 (10.2)			123 (12.4)			2 (6.5)
HER2		1.99	.22		0.85	.35	
+	384 (13.7)			93 (11.8)			3 (10)
−	2425 (86.3)			699 (88.2)			27 (90.3)
CH		96.2	<.01		0.01	.95	
y	1092 (35.2)			447 (53.9)			17 (51.6)
n	2010 (64.8)			382 (46.1)			14 (48.4)
Surgery		45.8	<.01		0.79	.27	
y	2602 (83.9)			873 (92.9)			31 (100)
n	489 (16.1)			67 (7.1)			0
Subtype		0.50	.45		0.25	.85	
HR+/HER2−	2354 (83.9)			606 (82.9)			23 (79.3)
HR+/HER2+	362 (12.9)			101 (13.8)			5 (17.2)
HR−/HER2+	22 (0.9)			7 (0.9)			0
HR−/HER2−	65 (2.3)			17 (2.3)			1 (3.4)
HG		18.9	<.01		2.13	.19	
I–II	1178 (65.1)			311 (76.2)			20 (64.5)
III	634 (34.9)			97 (23.8)			11 (35.5)
TNM		3.59	.06		1.29	.31	
(0–II)	1861 (75.2)			485 (71.6)			24 (77.4)
(III–IV)	613 (24.8)			192 (28.4)			7 (22.6)
Node		27.1	<.01		0.59	.45	
+	381 (41.0)			358 (30.2)	7		10 (37)
−	547 (59.0)			828 (69.8)			17 (63)

++ *X*^2^ and ^++^P = Chi-square and *P* value of data from SEER and East Asian; ^*+^*χ*^2^ and ^*+^*P* = Chi-square and *P* value of data from East Asian and JPH.

HG = histologic grade, JPH = Jiangsu Provincial Hospital, PR = progesterone receptor.

Table 4 also reveals that between East Asian and SEER patients, statistically significant differences were observed in some indicators (*P* < .05). Differences in CH, surgery, histological grade, and lymph node positivity were also recorded. For example, the proportion of East Asian patients receiving CH and surgery (53.9% and 92.9%, respectively) was higher than that of SEER patients (35.2% and 83.9%, respectively), and the proportion of East Asian patients with histological grades I to II (76.2%) was greater than that of SEER patients (65.1%). In terms of lymph node positivity, the proportion of East Asians (30.2%) was lower than that of SEERs (41%). Furthermore, no statistically significant difference was observed between the JPH and East Asian patients (all *P* > .05).

### 3.2. Correlation and regression analysis

Univariate analysis by the *χ*^2^ test was used on the indices of MBC patients in JPH, and the results showed a positive Pearson correlation between the lymph node (LN_1_), tumor size (TS_1_), and histologic grade (HG_1_), with *P* < .01, as shown in the left half of Table 5. Meanwhile, ER, PR, and HER2 statuses were not associated with lymph node-positive status (*P* > .05).

**Table 5:** Correlation and regression.

JPH correlation		Tj	HGj	SEER correlation		Ts	HGs
	Nj	0.809*	0.662*		Ns	0.298*	0.163*
	*P*	.000	.000		*P*	.000	.000
JPH regression				SEER regression			
Nj^e^	Beta	0.639	0.307	Ns^e^	Beta	0.277	0.111
	t	4.912	2.359		t	14.096	5.654
	*P*	.027	.000		*P*	.000	.000

E = dependent variable, HG = histologic grade, JPH = Jiangsu Provincial Hospital, SEER = surveillance, epidemiology, and end results.

* At the 0.01 level, the correlation is significant.

The above factors (*P* < .1 were used in the regression analysis, and the results showed a linear regression relationship between LN_1_, TS_1_, and HG_1_. LN_1_ is the dependent variable, and TS_1_ and HG_1_ are the independent variables. Thus, lymph node positivity increased with tumor size and histological grade, as shown in the left half of Table 5.

Similarly, in the SEER database, univariate analysis using the chi-square test revealed a correlation between positive lymph nodes (LN_2_), tumor size (TS_2_), and HG_2_, with *P* < .01, as shown in the right half of Table 5.

These factors were used for regression analysis, and the results showed a linear regression relationship among LN_2_, TS_2_, and HG_2_. LN_2_ was the dependent variable, and TS_2_ and HG_2_ were the independent variables. Thus, lymph node positivity increased with tumor size and histological grade, as shown in the right half of Table 5.

Notably, in patients with JPH and SEER, the same relationship existed; that is, lymph node-positive status increased correspondingly with tumor size and histological grade. A similar study on East Asian patients was not possible because data on MBC tumor size in the East Asian literature were unavailable.

## 4. Discussion

This study retrospectively analyzed and compared the clinicopathological data of patients with MBC from the JPH, East Asia, and SEER databases. Correlation and regression analyses were performed.

Several studies have reported the average age of MBC patients. For example, Wu pointed out that the average age of MBC patients in a hospital in Zhejiang Province, China (63.2 years) was smaller than that of patients from SEER (67.3 years).^[3]^ Literature reports that the average age of MBC patients in Hong Kong is 64.5 years.^[21]^ The results of this study showed that the average age of MBC patients from JPH and East Asia was lower than that from the SEER database. Therefore, the results of this study were consistent with those reported in the literature.

ER and PR expression has been reported in the literature^[3]^; the positive rates of ER and PR in MBC in a hospital in Zhejiang Province, China, were both 94.4%, and the positive rates of ER and PR in SEER were 96.6% and 89.6%, respectively. The results of this study showed that the positivity rates of ER and PR in JPH were 96.7% and 93.5%, respectively. In East Asian patients, ER had a positivity rate of 93.3%, and the value for PR was 87.7%. These findings indicate that the results of this study are similar to those reported in the literature.

Regarding the HER2− positive rate of MBC, Shao reported that the HER2− positive rate of MBC in Beijing, China was 10%^[14]^; another joint study of 93 centers in 9 countries reported that the HER2− positivity rate was about 8.6%.^[23]^ Wang reported that the HER2− positive rate in SEER patients was 12.1%.^[24]^ The results of this study showed that the positive rates of HER2 in patients from JPH, East Asia, and SEER were 9.7%, 11.7%, and 13.7%, respectively. Therefore, the results of this study on HER2 were similar to those reported in the existing literature.

Reports on the acceptance of CH and surgery in MBC patients vary. The proportion of MBC patients who received CH and surgery in Anhui Province, China, was 52% and 92%, respectively.^[10]^ Park reported that the proportion of Korean patients with MBC receiving CH and surgery was 56.2% (471/838) and 80.5%, respectively.^[25]^ Yao reported that 95.2 proportion of patients underwent surgery for SEER (2010–2014).^[26]^ The data from this study showed that the proportions of East Asian patients receiving CH and surgery were 51.6% and 100%, respectively. The proportions of CH and surgery in the SEER group were 35.2% and 83.9%, respectively. Compared with SEER MBC patients, East Asian MBC patients received CH and surgery at a higher rate. Is this due to the East Asian culture or the medical level and habits of doctors? Whether this affects the overall survival of East Asian patients remains to be studied in the future. The authors of this article consider that this has something to do with traditional Chinese culture. Because most Chinese people are very afraid of the word “cancer,” it seems that it is closely related to death.

Liu reported that the proportion of patients with HR+/HER2− in Tianjin, China was 80.3%,^[27]^ and another study reported that the proportion of patients with HR+/HER2− in Zhejiang, China was 83.3%,^[3]^ therefore, the results of this study on patients from JPH, East Asian, and SEER are similar to those in previous studies.

Hong reported that the proportion of HGs I to II in Korean MBC patients was 56.3%,^[22]^ and Wang mentioned that the proportion of HGs I to II in SEER was 61.34%,^[24]^ which is consistent with the results of this study on JPH and SEER patients.

Sun pointed out that the lymph node-positive ratio of MBC patients in Zhengzhou, China was 36.4%,^[20]^ Yao reported that the lymph node-positive ratio in SEER (2010–2014) was 43.1%,^[26]^ and Wachtel showed that the proportion of MBC with lymph node-positive status in SEER (1988–2003) was 34.6%.^[28]^ The proportions of lymph node-positive status in the JPH and SEER groups in this study were similar to those observed in other studies. However, the lymph node positivity ratio in East Asian patients was lower (30.2%), and this finding was observed possibly because the East Asian literature collected in this study contained data from the early days, that is, before 2014, and the test level at that time might have been low.^[29]^

Yang et al reported that tumor location, T stage, histological grade, and HER2 status were correlated with lymph node positivity.^[30]^ The results of this study showed a linear regression relationship between lymph node-positive status, tumor size, and histological grade in the JPH and SEER databases. In other words, lymph node positivity increased with tumor size and histological grade. Clearly, the results of this study go further than those of Yang because they find a numerical correlation.

Liu considered that the histological types of MBC included invasive MBC, MBC in situ and metastatic carcinoma.^[31]^ The invasive MBC was usually unilateral. The most common sign was a lump under the areola. The histological type and grade of invasive MBC were the same as the female. About 95% of the patients were AR positive, and ER and PR of most patients were positive. The positive rate of HER2 of the male was lower than that of the female. Generally, invasive lobular carcinoma of the male did not occur. The histological characteristics of the MBC in situ were similar to that of the female. Papillary ductal carcinoma in situ of the male breast was the most common type. Lobular tumor of the male was extremely rare.

With regard to MBC pathological differences in the different ethnic groups, Parise reported that black men with both low and high levels of concomitant disease had an increased risk of mortality when compared with white men with breast cancer.^[32]^ And, Ellington et al pointed out that relative survival 1 year after breast cancer diagnosis was lower among black males than it was among White and Hispanic males^[33]^; and a larger proportion of cases in Black males were diagnosed at distant stage than those in White and Hispanic males.

Reddington et al considered that factors playing a role in the incidence of the cancer in different ethnic groups included age, regions and pesticide^.[34]^ and, O’Malley et al expressed that the factors included gene and obesity.^[35]^

This study collected and analyzed the pathological data of East Asian MBC patients from 20 pieces of literature to solve the dilemma of the lack of specimens of East Asian MBC patients and determine the pathological truth of East Asian MBC. In this study, the data processing for each indicator was a simple arithmetic mean calculation. To compare the data between these groups, the *χ*^2^ test was performed. Differences were considered statistically significant at *P* < .05. This statistical method and analysis of the pathological data of East Asian MBC in the literature has a certain value in the current situation of a few East Asian MBC patients.

This study has the following limitations: Some of the data in the East Asian literature collected in this study are incomplete; and although the literature in this paper was collected from 2014 to 2021, several data were dated before 2014. The poor level of detection at that time may have affected the comparison results. These limitations should be addressed in future studies. And, it will be worthwhile for us to differentiate the cases based on different races from SEER to see whether races or lifestyle influence the clinicopathological features of MBC.

As a rare tumor, MBC lacks clinical research data, and its optimal treatment mode remains unclear. At present, clinical studies on East Asian MBCs are lacking. Therefore, the findings of this study have implications to deepen our understanding of East Asian MBC.

## Author contributions

**Conceptualization:** Lei Xi.

**Data curation:** Jinxing Zhou.

**Formal analysis:** Yan Wu.

**Writing – review & editing:** Rong Rong.

## Supplementary Material


