# Supplementary figures and images for: Clinicopathological features and correlation analysis of male breast cancer
Source: Medicine (Baltimore). 2023 Jul 28;102(30):e34408. doi: 10.1097/MD.0000000000034408 (PMC10378966; doi:10.1097/MD.0000000000034408)

[Supplementary Figure S1]

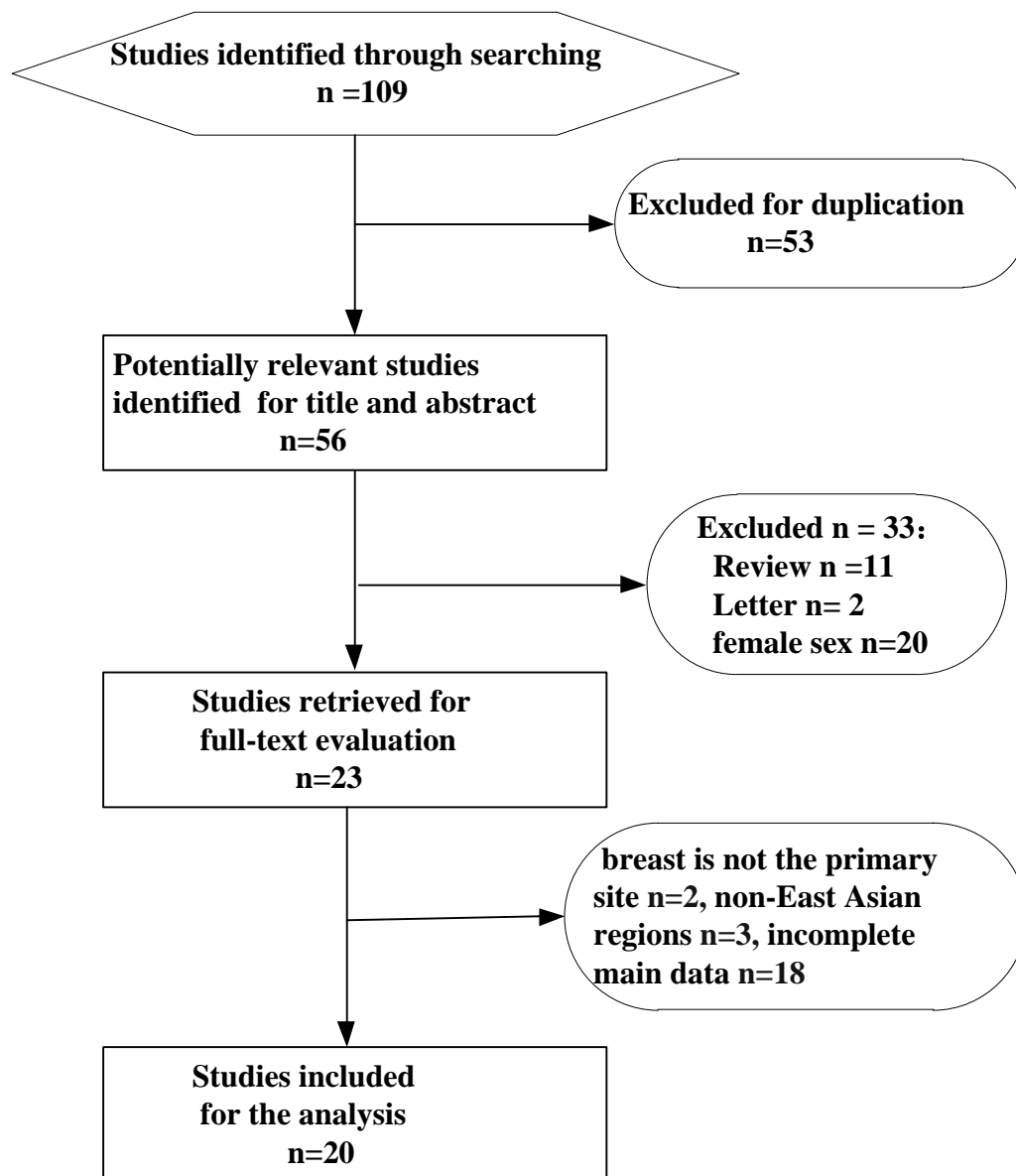

Supplementary Figure S1. Article selection process

Supplement: Supplementary file 1 [file medi-102-e34408-s001.pdf]
